# Rare Ganglion Cyst of the First Metatarsophalangeal Joint: A Case Report

**DOI:** 10.7759/cureus.95087

**Published:** 2025-10-21

**Authors:** Yuto Yamamura, Kazuyasu Fujii, Maiko Kato, Sho Onishi, Atsushi Otsuka

**Affiliations:** 1 Dermatology, Kindai University, Osaka, JPN; 2 Dermatology, Kindai University Hospital, Osaka, JPN; 3 Dermatology, National Hospital Organization Osaka Minami Medical Center, Osaka, JPN

**Keywords:** dermatologic surgery, foot, ganglion cyst, intraoperative diagnosis, metatarsophalangeal joint

## Abstract

Ganglion cysts are common benign lesions arising from joint capsules or tendon sheaths, but their occurrence in the first metatarsophalangeal (MTP) joint is extremely rare. Preoperative diagnosis of subcutaneous tumors in this region can be difficult, as imaging findings are often nonspecific and biopsy may be avoided due to the risk of nerve or vessel injury. We report the case of a woman in her 70s who presented with a slowly enlarging subcutaneous mass on the medial aspect of the right first MTP joint. MRI and ultrasound suggested a cystic lesion but failed to confirm continuity with the joint capsule. During surgery, jelly-like contents were identified, confirming the diagnosis of a ganglion cyst. Complete excision including the stalk was performed, and no recurrence has been observed after six months of follow-up. This case demonstrates that intraoperative findings can be crucial for establishing a diagnosis and ensuring complete excision when preoperative evaluation is inconclusive. Dermatologists performing minor surgery should recognize the importance of intraoperative assessment when managing diagnostically challenging subcutaneous tumors.

## Introduction

Ganglion cysts are common benign lesions encountered in daily clinical practice; however, their imaging characteristics often resemble those of peripheral nerve sheath tumors and hemangiomas, making differential diagnosis challenging [1,2]. Moreover, even with advanced imaging modalities such as MRI, a definitive preoperative diagnosis cannot always be established in a subset of cases [3]. In addition, biopsy of subcutaneous tumors with uncertain diagnosis is frequently avoided due to the risk of nerve injury or bleeding, and surgery may therefore proceed without histological confirmation [4].

Ganglion cysts most commonly occur on the dorsal wrist, whereas only about 10% develop in the foot, and those arising in the toes are even rarer [5]. Among them, cysts localized around the first metatarsophalangeal (MTP) joint are extremely uncommon [6], and preoperative diagnosis can be particularly challenging in such rare locations.

We report a rare case of a ganglion cyst arising from the medial first metatarsal region, presenting with an atypical location of the cystic mass. Preoperative imaging failed to demonstrate continuity with the first MTP joint, rendering the diagnosis uncertain. However, intraoperative findings confirmed the diagnosis of a ganglion cyst, which enabled the selection of an appropriate surgical approach. This case highlights the clinical significance of intraoperative assessment in guiding both diagnosis and treatment strategy for subcutaneous tumors that are difficult to characterize preoperatively, particularly when imaging findings are inconclusive.

## Case presentation

**Figure 1:**
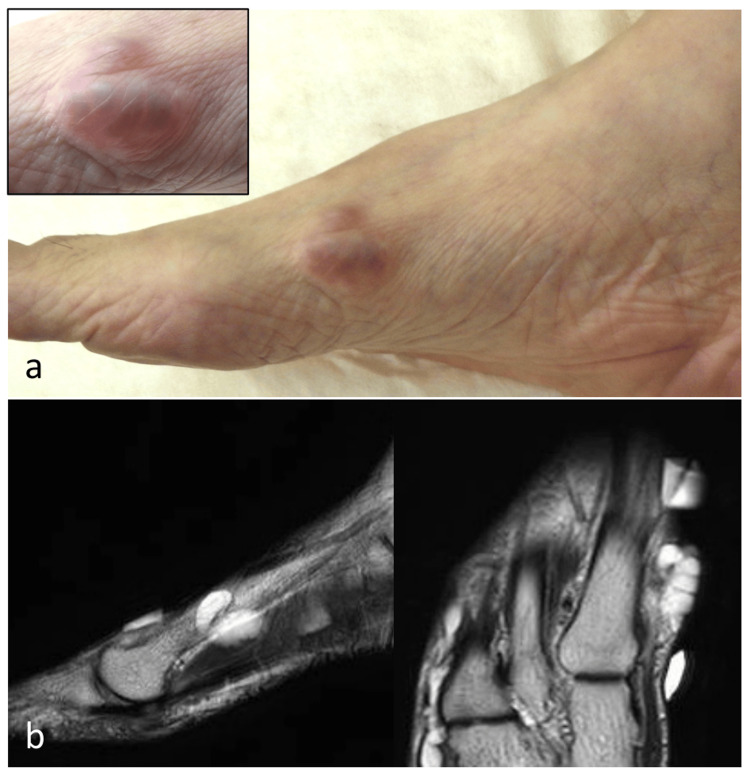
Clinical photograph and MRI findings a. A subcutaneous mass with elastic hardness and mobility over the underlying tissue is observed on the medial aspect of the first metatarsal. The lesion is visible beneath the skin and was associated with mild tenderness. b. Preoperative T2-weighted MRI shows a multilocular cystic lesion with high signal intensity; however, clear continuity with the first metatarsophalangeal joint was not identified.

A woman in her 70s presented with a subcutaneous mass on the medial side of her right foot, which she had first noticed five to six years earlier. The mass had gradually enlarged and was intermittently associated with mild pain, prompting consultation at our department. On physical examination, a firm, elastic, and mobile subcutaneous tumor was identified over the medial aspect of the first metatarsal (Figure 1A).

Magnetic resonance imaging (MRI) revealed a well-defined, multilocular cystic lesion with low signal intensity on T1-weighted images and high signal intensity on T2-weighted images; however, clear continuity with the first MTP joint was not demonstrated (Figure 1B). Ultrasonography showed no internal vascularity. Differential diagnoses included hemangioma, schwannoma, and ganglion cyst. Considering the risk of nerve injury and bleeding, biopsy was avoided, and complete excision was planned.

**Figure 2:**
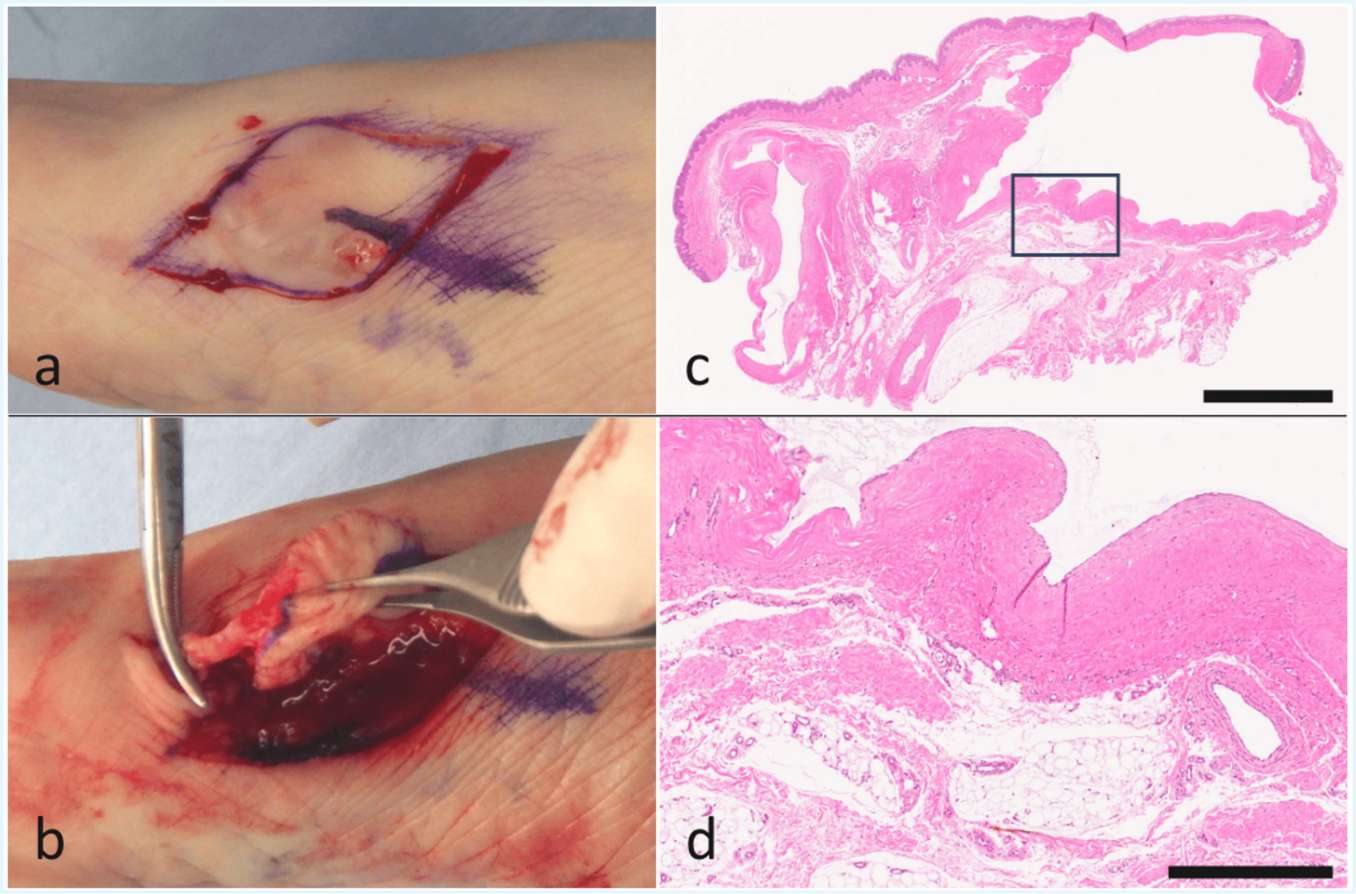
Intraoperative and histopathological findings a. After skin incision, a soft, elastic mass with translucent contents was identified. Partial incision of the mass revealed gelatinous material, leading to the intraoperative diagnosis of a ganglion cyst. b. Careful dissection revealed a stalk extending toward the capsule of the first metatarsophalangeal (MTP) joint, which was ligated and resected at its base. c. Low-power photomicrograph (H&E stain) showing a multilocular cystic lesion surrounded by fibrous capsule. The black box indicates the region shown in panel d. Scale bar: 2.5 mm. d. High-power photomicrograph (H&E stain) demonstrating a fibrous cyst wall lacking an epithelial lining, with mucoid material in the cystic cavity. Scale bar: 500 µm.

The skin incision was designed to allow access compatible with both ganglion cyst and schwannoma. After incision, a soft, elastic tumor containing translucent fluid was identified. When a portion was opened, a jelly-like material was expressed (Figure 2A), leading to the intraoperative diagnosis of ganglion cyst. Careful dissection revealed a stalk extending toward the first MTP joint capsule, which was traced to its base, ligated, and resected (Figure 2B). Histopathological examination confirmed the diagnosis of ganglion cyst (Figure 2C, 2D). The postoperative course was uneventful, and no recurrence was observed at the six-month follow-up.

## Discussion

Ganglion cysts are benign cystic lesions arising from joint capsules or tendon sheaths. They are most frequently observed on the dorsal wrist, while only about 10% occur in the foot [5]. Occurrence in the toes is even rarer, and cases localized to the first MTP joint are extremely uncommon [6]. The first MTP joint bears significant mechanical stress during walking and toe-off [6], and it is also subject to repetitive compression from footwear, factors that have been reported as potential contributors to the development of ganglion cysts [7]. Such mechanical factors may have played a role in the present case. Because of the rarity of ganglion cysts in this location, describing the clinical course and diagnostic process in detail may help clinicians recognize and appropriately manage similar lesions in the future.

On MRI, ganglion cysts typically appear as cystic lesions; however, continuity with the adjacent joint is not always demonstrable, and differentiation from other lesions such as hemangiomas and schwannomas can be challenging [3]. In addition, biopsy of tumors located near peripheral nerves or vessels is often avoided due to the risk of complications such as nerve injury or hemorrhage [4]. In the present case, the cystic mass was located along the medial aspect of the first metatarsal, making it difficult to visualize its continuity with the MTP joint on preoperative imaging. Nevertheless, intraoperative identification of gelatinous contents and a stalk continuous with the joint capsule confirmed the diagnosis of a ganglion cyst and enabled curative resection. The separation of the cystic body from the stalk is considered a characteristic feature of ganglion cysts [7], and this case demonstrated such a typical morphology.

It is well established that simple excision of the cyst alone carries a high risk of recurrence, as the cystic body and the communication stalk with the joint capsule or tendon sheath often exist apart from each other. In contrast, resection including the stalk has been reported to prevent recurrence [8]. In this case, complete excision including the base of the stalk likely contributed to the absence of recurrence to date.

This case emphasizes the importance of intraoperative assessment in achieving an accurate diagnosis and curative resection of subcutaneous cystic lesions occurring in atypical locations.

## Conclusions

This case represents an extremely rare ganglion cyst of the first MTP joint. Although preoperative imaging did not allow definitive diagnosis, intraoperative findings facilitated both accurate diagnosis and curative excision. Even in subcutaneous tumors where preoperative diagnosis is difficult and biopsy may be avoided, intraoperative findings can play a crucial role in determining diagnosis and guiding surgical strategy, thereby enabling safe and recurrence-free treatment. Beyond this individual case, such intraoperative assessment can provide valuable guidance for clinicians managing subcutaneous lesions of uncertain nature, emphasizing its practical and educational importance in daily clinical practice.
